# Anticancer Activity In Vitro of Sulfated Polysaccharides from the Brown Alga *Spatoglossum vietnamense*

**DOI:** 10.3390/molecules29214982

**Published:** 2024-10-22

**Authors:** Dinh Thanh Trung, Valerii Victorovich Surits, Anastasia Olegovna Zueva, Hang Thi Thuy Cao, Natalia Michailovna Shevchenko, Svetlana Pavlovna Ermakova, Pham Duc Thinh

**Affiliations:** 1 Nhatrang Institute of Technology Research and Application, Vietnam Academy of Science and Technology, Nha Trang 650000, Vietnam; dinhthanhtrung410@gmail.com (D.T.T.); caohang.nitra@gmail.com (H.T.T.C.); 2 G.B. Elyakov Pacific Institute of Bioorganic Chemistry, Far Eastern Branch of the Russian Academy of Sciences, 690022 Vladivostok, Russia; suritsw@yandex.ru (V.V.S.); a.o.zueva@yandex.ru (A.O.Z.); natalyshe@piboc.dvo.ru (N.M.S.)

**Keywords:** brown seaweed, colon cancer, DLD-1, fucoidan, HCT-116, HT-29, soft agar, Spatoglossum

## Abstract

Sulfated polysaccharides SpvF1, SpvF2, SpvF3, and SpvF4 from the brown alga *S. vietnamense* collected north of Hon Do (Nha Trang Bay, Vietnam) were isolated and studied. The structure of the obtained polysaccharide was studied using chemical methods and NMR spectroscopy. Fucoidans were low-sulfated (SpvF1, SpvF2) and medium-sulfated (SpvF3, SpvF4) heterogeneous polysaccharides. The molecular weight of the polysaccharides obtained was in the range of 16 to 44 kDa. All investigated fucoidans until 400 µg/mL were not cytotoxic for human colon carcinoma cells DLD-1, HCT-116, and HT-29 in vitro. Fucoidans SpvF1 and SpvF2 have inhibited the colony formation and growth of investigated cells from 20 to 30%. Fucoidans SpvF3 and SpvF4 have the strongest inhibitory effect for investigated cancer cells: from 40 to 50%.

## 1. Introduction

Many algae, which belong to different classes—brown, red, and green—grow in the coastal waters of Vietnam [1]. They are used in traditional medicine, agriculture, and cosmetics because algae contain various compounds that have beneficial properties. A comprehensive study of the potential of algae is important from the applied and scientific sides. The most widely represented algae in Vietnam are those of the Sargassum genus. The brown alga *S. vietnamense* belongs to the order Dictyotales, the family Dictyotaceae, genus Spatoglossum. The Spatoglossum genus grown in Vietnam includes *Spatoglossum schroederi*, *Spatoglossum stipitatum*, and *S. vietnamense*. *S. vietnamense* grows in the South China Sea near the coasts of Malaysia and Vietnam [1]. Brown algae are a source of various minerals. They also contain polyphenols and polysaccharides.

For the isolation of polysaccharides, classical extraction methods combined with supercritical fluid extraction, enzymatic extraction, etc., are used. The determination of the main structural characteristics (monosaccharide composition, molecular weight, and sulfate content) is carried out using various physicochemical methods. The study of biologically active compounds of brown algae as antioxidants, antidiabetic, and antitumor drugs arouses great interest [2,3,4]. The brown algae *Spatoglossum schroederi* and *Spatoglossum asperum* are the most studied in terms of content, structure, and biological activities of polysaccharides [5,6,7,8,9,10,11,12,13,14]. The sulfated polysaccharides have been isolated from the brown alga *S. schroederi*: fucan A, fucan B, and fucan C. All of them were galactofucans. Nanoparticles have been obtained based on fucan A and B. The biological effects of both native sulfated polysaccharides and nanoparticles have been investigated [5,10,11,12,13,14,15]. Sulfated polysaccharides from *S. asperum* are less studied [7,8]. There are no published data on the study of polysaccharides from the brown alga *S. vietnamense*.

The goal of the present work was to investigate and compare the structural characteristics and antitumor activity in vitro of sulfated polysaccharides from the brown alga *S. vietnamense* collected in Nha Trang Bay, Vietnam. These are the first data about the content, structure and biological activities of fucoidans from *S. vietnamense.*

## 2. Results

### 2.1. Isolation and Characterization of Polysaccharides

**Figure 1:**
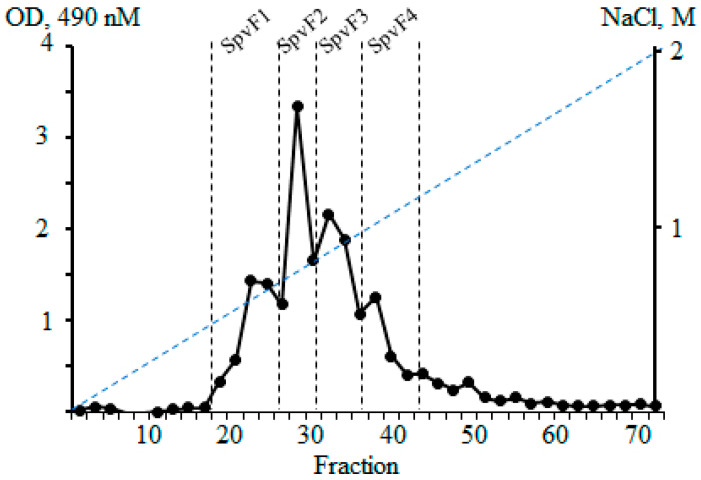
Ion-exchange chromatography on Macro-Prep DEAE (Cl^−^ form, 8 × 2.5 cm) of water-soluble polysaccharides from *S. vietnamense*.

Polysaccharides from brown algae were isolated according to the procedures described in Materials and Methods. As a result of the fractionation of polysaccharides with a linear gradient of NaCl on an anion exchange carrier Macro-Prep DEAE, fractions of sulfated polysaccharides (fucoidans, F) were isolated from the brown algae *S. vietnamense* SpvF1, SpvF2, SpvF3, and SpvF4 (Figure 1) with yields 0.42, 0.41, 0.75, and 0.56%, respectively.

The structural characteristics of the polysaccharides obtained have been studied (Table 1). The analysis of the monosaccharide composition of the polysaccharides that we isolated from *S. vietnamense* has shown that they are all heteropolysaccharides (Table 1). The main monosaccharide residue of the SpvF1 and SpvF2 fractions obtained by anion-exchange chromatography of the polysaccharide SpvP is mannose; SpvF3 and SpvF4—fucose, mannose, and xylose. Low-sulfated SpvF1, SpvF2 (3.5 and 5.5%), and medium-sulfated SpvF3 and SpvF4 (10.4 and 8.8%) polysaccharides were obtained. The molecular weight of the polysaccharides obtained was in the range of 16 to 44 kDa.

The heterogeneous structure of the fucoidans isolated from *S. vietnamense* has been confirmed by ^13^C NMR spectroscopy data (Supplementary Figure S1). As is the case with many other fucoidans from brown algae, the ^13^C NMR spectra of the fucoidans studied are complex and not very informative for structural analysis. However, they contain groups of signals in the anomeric region (96–104 ppm), as well as signals typical for α-L-fucopyranosides in the region of high-frequency fields (16.5–16.9 ppm). Signals at 21–22 ppm (CH_3_) and 175–176 ppm (C=O) indicated the presence of acetyl groups was not registered in the ^13^C NMR spectra of the investigated fucoidans.

### 2.2. Biological Activity of Polysaccharides

Sulfated polysaccharides from various brown algae are known to exhibit antitumor effects in various in vitro systems. However, there are no publications in the literature studying the antitumor effect of polysaccharides from Spatoglossum.

Therefore, it was of interest to study the antitumor effect of sulfated polysaccharides from the South China Sea brown alga *S. vietnamense* on various cancer cells in vitro. Human colon carcinoma cells HCT-116, HT-29, and DLD-1 were chosen as a model. Previously, it was shown for fucoidans from genus Sargassum that galactofucans inhibited the formation of colonies of human colon carcinoma cells [16].

This study of the biological activity of the substances at the first stage involves the assessment of their toxicity. Treatment of HCT-116, HT-29, and DLD-1 cells with the studied sulfated polysaccharides at a concentration of up to 400 µg/mL did not lead to inhibition of their growth and was not accompanied by mass cell death. Consequently, the investigated polysaccharides at a concentration of up to 400 µg/mL are not toxic for HCT-116, HT-29, and DLD-1 cells (Supplementary Figure S2).

**Figure 2:**
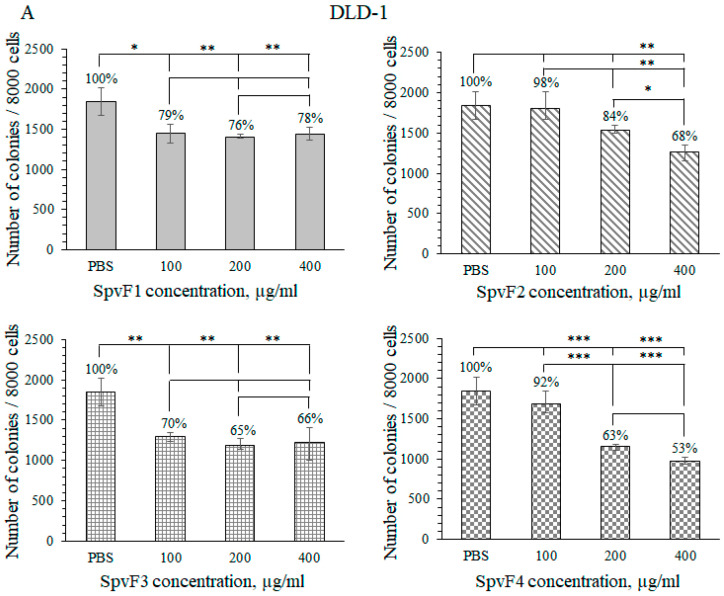

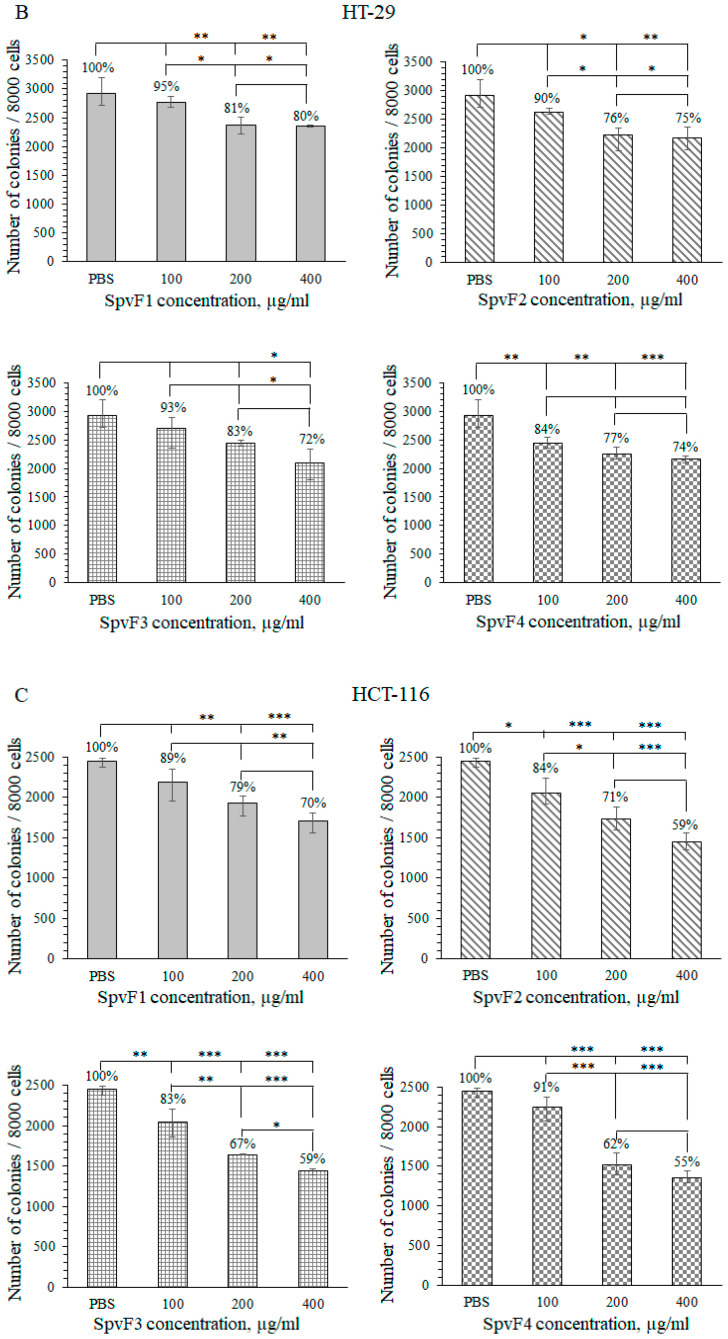
The inhibitory effects of polysaccharides SpvF1, SpvF2, SpvF3, and SpvF4 (100–400 μg/mL) on colony formation of human colorectal adenocarcinoma cell lines DLD-1 (**A**), HT 29 (**B**), and HCT-116 (**C**). Results are expressed as the mean ± standard deviation (SD). The asterisk (*) indicates a significant decrease in colonies number treated by polysaccharides compared with control (* *p* < 0.05, ** *p* < 0.01, *** *p* < 0.001).

Then, we investigated the activity of fucoidans SpvF1, SpvF2, SpvF3, and SpvF4 against colony formation of cancer cells HCT-116, HT-29, and DLD-1 in vitro (Figure 2).

The sulfated polysaccharide SpvF1 (400 µg/mL) showed weak inhibition of colony formation and growth of all investigated cells: for DLD-1—22%, HT-29—20%, and for HCT-116—30%. For the sulfated polysaccharide SpvF2, the degrees of inhibition were for DLD-1—32%, HT-29—25%, and for HCT-116—41%. Fucoidan SpvF3 inhibited the colony formation and growth of DLD-1 cells by 44%, HT-29 by 28%, and for HCT-116 by 41%. Fucoidan SpvF4 inhibited colony formation of DLD-1 and HCT-116 cells by almost 50%, and inhibition of HT-29 cell colony formation and growth was 26%. Thus, fucoidans SpvF3 and SpvF4 have the strongest inhibitory effect for colony formation and growth of cancer cells. So, we have shown that sulfated polysaccharides SpvF1 and SpvF2 were less active than sulfated polysaccharides SpvF3 and SpvF4.

## 3. Discussion

Sulfated polysaccharides of brown algae—fucoidans—attract the attention of researchers because they have various biological activities. It is important to find new sources of fucoidans. The content of fucoidans in brown algae of different families varies. In the algae of the genus Sargassum, collected in the coastal waters of Vietnam, the content of fucoidans is low, less than 1%. These algae synthesized heterogeneous sulfated fucoidans [16]. As was published, the yield of fucoidans from *S. asperum* was 4.4% [7], which is not typical for brown algae growing in warm waters. Perhaps the authors meant the yield of the fraction obtained after the extraction of polysaccharides. The total yield of fucoidans obtained by us from *S. vietnamense* was 2.14% of the dry alga weight. The yields of fucoidans after fractionation were 0.42, 0.41, 0.75, and 0.56% for SpvF1, SpvF2, SpvF3, and SpvF4, respectively.

Sulfated polysaccharides of brown algae, fucoidans, are divided into fucans—fucoidans built mainly from fucose residues; galactofucans—fucoidans in which the main monosaccharide residues are fucose and galactose; and heterogeneous fucoidans, the monosaccharide composition of which can be represented by fucose, galactose, mannose, xylose, rhamnose, and glucuronic acid. Fucoidans from *S. schroederi* and *S. asperum* are known to be heterogeneous in monosaccharide compositions. The main chain of fucan A from *S. schroederi* is built from β-(1,3)-linked glucuronic acid with branches of α-(1,3)-linked fucose chains. Fucose residues are substituted at C4 and C2 (minor) with sulfate groups. Also, fucose residues are substituted at C2 with partially sulfated chains of β-(1,4) xylose [11]. The main chain of fucan B is built from residues of fucose (1 mol%), galactose (2 mol%), and xylose (0.5 mol%). The content of glucuronic acid was 0.1 mol%. The amount of sulfate was 19%. The main chain of fucoidan from *S. asperum* contained residues of fucose (60.9%), galactose (25.2%), mannose (4.2%), rhamnose (3.4%), and xylose (6.3%). The amount of sulfate was 21% [7]. The fucoidans isolated from the brown alga *S. vietnamense* collected in the coastal waters of Vietnam were also heterogeneous in the monosaccharide composition. They contained residues of fucose, galactose, mannose, xylose, and glucose in various proportions. The content of sulfates was from 3.5 to 10.4%. Our data confirmed that brown algae growing in warm waters synthesize heterogeneous fucoidans.

It is well known that the sulfated polysaccharides isolated from algae have a wide range of biological activities. The literature provides information on the biological effects of fucoidans isolated from the algae *S. schroederi* and *S. asperum*. It has been shown that the brown alga *S. schroederi* (Dictyotaceae) contains three main types of sulfated polysaccharides (fucans A, B, and C) [10]. These polysaccharides contain different molar ratios of fucose, xylose, galactose, and sulfate. Nanoparticles based on these polysaccharides were also created, and their effect was studied in comparison with the effect of native polysaccharides.

It was shown that fucan A from *S. schroederi* stimulates the synthesis of antithrombotic heparan sulfate from endothelial cells like heparin. The hypothesis has been raised that the in vivo antithrombotic activity of fucan A is related to the increased production of this heparan. Taken together with the fact that the compound is practically devoid of anticoagulant and hemorrhagic activity, the data suggest that it may be an ideal antithrombotic agent in vivo [5]. Fucan B from *S. schroederi* has no anticoagulant activity but strongly stimulates the synthesis of heparan sulfate by endothelial cells. We suggested that this last effect may be related to the in vivo antithrombotic activity of this polysaccharide [13]. Fucan C (xylogalactofucan) has neither anticoagulant (from 10 to 100 µg/mL) nor hemorrhagic activities (100 µg/mL) [10].

The activity of sulfated polysaccharides from *S. schroederi* was investigated in different models in vitro. Fucan A (up to 100 µg/mL) showed no cytotoxicity against human renal adenocarcinoma (786-0) cells (ATCC CRL-1932), murine melanoma (B16F10) cells (ATCC CRL-6475), human renal cells (HEK-293) (ATCC CRL-1573), murine fibroblasts cells (3T3) (ATCC CCL-92), canine kidney cells (MDCK), monkey kidney cells (Vero cells), Chinese hamster ovarian cells (CHO-K1), human uterine cervical carcinoma cells (SiHa), and human liver carcinoma cells (HepG2) (20). Fucan A exhibited antitumor effects in in vitro models. Polysaccharides modulate gene expression and reduce the proliferation rate and tube formation of CHL-1 (tripleWT) human melanoma cells. They also reduce the cell density of CHL-1, similar to that of cisplatin (57% and 61%, respectively) [9]. It should be noted that silver nanoparticles containing fucan A (AgFuc) (up to 100 µg/mL) induce death of renal adenocarcinoma 786-0 and murine melanoma (B16F10) [15].

Another polysaccharide from *S. schroederi*—fucan B (sulfated galactofucan) (0.012–0.1 µg/mL) inhibited endothelial cells (ECs) capillary-like tube formation and migration in a concentration-dependent manner but did not affect ECs proliferation at a range of concentrations 0.01–0.05 µg/mL. It was shown that biotinylated fucan B did not bind to the cell surface but rather only to fibronectin [6]. Fucan B inhibited adhesion to fibronectin in CHO-K1 cells (wild-type) and adhesion to type I collagen in CHO-745 cells (deficient in xylosyltransferase) [11].

Polysaccharides from *S. schroederi*—fucan A displayed strong antigenotoxic activity against oxidizing chemicals (H_2_O_2_) but not against alkylating chemicals (MMS) [14].

Silver nanoparticles containing fucan A (AgFuc) were shown to be more effective in inhibiting the ability of parasites to reduce MTT than fucan A or silver, regardless of treatment time. Therefore, AgFuc induces damage to the parasites’ mitochondria, which suggests that it is an anti-Trypanosoma cruzi agent [17].

Fucoidan from another brown alga, *S. asperum*, exhibited antioxidant effects (dose-dependent) and antibacterial potential (100 μg/mL) against *Aeromonos hydrophila* [7].

The silver nanoparticles (Fu-AgNPs) synthesized with fucoidan from *S. asperum* showed potential antibacterial activity against *Klebsiella pneumoniae* in agar bioassay, disk diffusion, reactive oxygen species, protein leakage, and confocal laser scanning microscopy assays [8]. Determination of the relationship between structure and biological activity for the polysaccharides from *S. schroederi* and *S. asperum* is difficult since they are heterogeneous.

In this work, the antitumor effect of sulfated polysaccharides from the South China Sea’s brown alga *S. vietnamense* on various cancer cells in vitro was studied. The investigated fucoidans (up to 400 μg/mL) were non-toxic against human colon carcinoma cells DLD-1, HCT-116, and HT-29. It should be noted that fucan A was also non-toxic (up to 100 μg/mL) towards various cell types [18]. The fucoidans SpvF1, SpvF2, SpvF3, and SpvF4 from *S. vietnamense* differed in the monosaccharide composition and degree of sulfation. It is known that the degree of sulfation plays an important role in the biological activity of fucoidans. Low-sulfated fucoidans SpvF1 and SpvF2, containing more than 50% mannose, inhibited colony formation from 20 to 30%. Medium-sulfated fucoidans SpvF3 and SpvF4, in which the sulfate content was up to 10%, inhibited colony formation by 40-50% (HCT-116 and DLD-1 cells). Our data confirm that with increasing sulfate content, the biological activity of fucoidans increases.

The search for new sources of sulfated polysaccharides—fucoidans—is important from the scientific and practical sides. Purification of new fucoidans and determination of their structural characteristics expands the information on the structural classes of fucoidans. This study of the biological activity of structurally characterized fucoidans will allow us to accumulate a volume of experimental data to determine the relationship between structure and biological activity. The most active were fucoidans SpvF3 and SpvF4. We have shown that the brown alga *S. vietnamense* is a new source of biologically active fucoidans.

## 4. Materials and Methods

### 4.1. Materials

Organic solvents, inorganic acids and salts, sodium hydroxide, trifluoroacetic (TFA), and trichloroacetic (TCA) acids were commercially available (Laverna-Lab, Moscow, Russia). Standards (mannose, rhamnose, glucose, galactose, xylose, and dextrans), phosphate-buffered saline (PBS), L-glutamine, penicillin–streptomycin solution (10,000 U/mL, 10 μg/mL) were purchased from Sigma-Aldrich (St. Louis, MO, USA). The Basal Medium Eagle (BME), McCoy’s 5A Modified Medium (McCoy’s 5A), and Roswell Park Memorial Institute medium (RPMI-1640), trypsin, and fetal bovine serum (FBS) were purchased from ThermoFisher Scientific (Waltham, MA, USA). MTS reagent—3-[4,5-dimethylthiazol-2-yl]-2,5-diphenyltetrazolium bromide was purchased from Promega Corporation (Madison, WI, USA).

The sorbent for chromatography, Macro-Prep DEAE, was obtained from Bio Rad Laboratories, Inc. (Hercules, CA, USA).

The brown alga *S. vietnamense* (Spv) was collected north of Hon Do (Nha Trang Bay, Vietnam) in April 2023. Next, the fresh alga was washed with seawater and soaked in 96% ethanol for 10 days to remove lipids and pigments. The defatted alga was air-dried at room temperature.

### 4.2. Procedures

#### 4.2.1. Polysaccharide Extraction

Samples of dried defatted alga (200 g) were extracted with HCl (0.1 M, 2 L) for 2 h at 60 °C. The extracts were neutralized and centrifuged. The supernatant was concentrated in vacuo, dialyzed against distilled H_2_O, and lyophilized to afford fractions containing fraction SpvP.

#### 4.2.2. Anion-Exchange Chromatography of Polysaccharides on Macro-Prep DEAE

The sample (1 g) of polysaccharide SpvP was dissolved in 20 mL of 0.04 M HCl and centrifuged. Then, the supernatant was applied to a Macro-Prep DEAE column (Cl^−^ form, 2.5 × 8 cm) equilibrated with 0.04 M HCl. First, the column was eluted with water, then with a linear gradient of NaCl (from 0.1 M to 2 M). All fractions were analyzed using the phenol–sulfuric acid method [19]. The fucoidan-containing (F) fractions were concentrated on a rotary evaporator, dialyzed, and lyophilized to obtain fractions SpvF1, SpvF2, SpvF3, and SpvF4.

#### 4.2.3. Acid Hydrolysis of Polysaccharides

A sample (5 mg) was dissolved in TFA (0.5 mL, 2 M), hydrolyzed at 100 °C for 6 h, neutralized with aqueous NH_4_OH, and evaporated to dryness.

### 4.3. Analyses

#### 4.3.1. Instruments

Nuclear magnetic resonance (NMR) spectra were obtained using an Avance DPX-500 NMR spectrometer (Bruker BioSpin Corporation, Billerica, MA, USA) at 35 °C. The concentration of the samples was 15 mg of polysaccharide/600 µL of D_2_O.

#### 4.3.2. Analytical Procedures

Total carbohydrates were quantified using the phenol–sulfuric acid method [19]. The monosaccharide composition was determined by gas–liquid chromatography after hydrolysis using 2M TFA (6 h, 100 °C) and obtaining the alditol acetate derivatives. The number of sulfate groups was determined using the BaCl_2_ gelatin method [20].

#### 4.3.3. Molecular Weight Determination

The molecular weights of polysaccharides were determined by high-performance size-exclusion chromatography (HPSEC) using the HPLC instrument Shimadzu LC-20 Series (Shimadzu, Kyoto, Japan) equipped with LC-20AD pump, degassing unit DGU-20A5R, autosampler SIL-20AHT, column oven CTO-20A, and refractive index detector RID-20A on the GPC column PSS SUPREMA combination ultrahigh (three columns, dimensions 8 mm × 300 mm, particle size 10 µm, PSS, Mainz, Germany). Elution was performed with Lithium nitrate (0.1 M) at 40 °C, with a flow rate of 1.0 mL/min. Different dextran standards (Sigma, Cibolo, TX, USA) of 1, 5, 12, 25, 50, 150, 270, and 670 kDa were used as reference standards.

#### 4.3.4. Biological Activity

Cell culture. The HT 29 (ATCC # HTB-38™), HCT-116 (ATCC # CCL-247™) human colon cancer cell lines were cultured in McCoy’s 5A medium; DLD-1 (ATCC # CCL-221™) human colon cancer cell line was maintained in RPMI-1640 medium (ThermoFisher Scientific, Waltham, MA, USA), respectively, supplemented with 10% (*v*/*v*) heat-inactivated FBS, 2 mM L-glutamine, and 1% penicillin–streptomycin in a humidified atmosphere containing 5% CO_2_.

Cell cytotoxicity assay. To estimate cytotoxicity, cells were seeded (3 × 10^4^) in 96-well plates in 200 μL of the appropriate medium and incubated at 37 °C in a 5% CO_2_ incubator. After 24 h, the medium was removed and replaced with fresh medium containing different concentrations (100, 200, and 400 μg/mL) of the fucoidan for an additional 24 h at 37 °C in a 5% CO_2_ incubator. After incubation, 3-(4,5-dimethylthiazol-2-yl)-5-(3-carboxymethoxyphenyl)-2-(4-sulfophenyl)-2H-tetrazolium inner salt (MTS reagent) (10 μL) was added to each well, and cells were incubated for 4 h at 37 °C and 5% CO_2_. Absorbance was measured at 490/630 nm.

Soft agar clonogenic assay. The soft agar assay was performed using HT-29, HCT-116, and DLD-1 cells. In brief, cells (2.4 × 10^4^/mL) were grown in 1 mL of 0.3% Basal Medium Eagle’s agar (ThermoFisher Scientific) containing 10% FBS. The culture was maintained at 37 °C in a 5% CO_2_ incubator for 2 weeks. Cell colonies were scored using a microscope (XiangAn, Xiamen, China) and ImageJ 1.45 software, as previously described [16].

Data analysis. All figures shown in this study are representative of at least three independent experiments with similar results. Statistical differences were evaluated using Student’s *t*-test and considered significant at *p* ≤ 0.05.

## 5. Conclusions

Sulfated polysaccharides were isolated from the brown alga *S. vietnamense* collected off the coast of Vietnam. The yield of sulfated polysaccharides was 2.14%: the polysaccharides SpvF1 and SpvF2 were heterogeneous in the monosaccharide composition, they had a significant amount of mannose residues (86.6 and 69.8%, respectively), and the total amount of other monosaccharide residues (Fuc, Gal, Xyl, Glc) was about 13% and 30%, respectively. The heteropolysaccharides SpvF3 and SpvF4 are fucoxylans, and the total amount of other monosaccharide residues (Gal, Man, Glc) was 41.4% and 43.7%, respectively. The sulfated polysaccharides SpvF3 and SpvF4 inhibited colony formation of human colon cancer cells in vitro. We have shown that the fucoidans from various brown algae, with their different structures, inhibited different types of cancer cells in vitro. It would be interesting to study in future investigations the anticancer activity of fucoidans SpvF3 and SpvF4 on other types of tumor cells and determine their specificity. In addition, it would be interesting to determine their effect on the metabolism of cancer cells.

## Figures and Tables

**Table 1:** Yields and structural characteristics of polysaccharides from *S. vietnamense*.

Fraction	Yield, % *	SO_3_Na, % **	Mw, kDa	Monosaccharide Composition, mol %				
				Fuc	Gal	Man	Xyl	Glc
SpvF1	0.42	3.5	16.266	3.6	0.8	86.6	5.3	3.6
SpvF2	0.41	5.5	25.266	9.3	2.4	69.8	11.5	6.8
SpvF3	0.75	10.4	31.931	22.8	7.5	20.3	35.7	13.6
SpvF4	0.56	8.8	44.047	24.3	17.4	8.9	31.8	17.4

Notes: * by dry defatted alga weight; ** by sample weight.

## Data Availability

Data are contained within the article and Supplementary Materials.

## Supplementary Materials

The following supporting information can be downloaded at: https://www.mdpi.com/article/10.3390/molecules29214982/s1, Figure S1: The C^13^ NMR spectra of fucoidans SpvF2, SpvF3, SpF4. Figure S2: The cytotoxicity of fucoidans SpvF2, SpvF3, SpF4 from *S. vietnamense* against colon cancer cells DLD-1 (A), HT-29 (B), and HCT-116 (C).
