# Supplementary material for: Anticancer Activity In Vitro of Sulfated Polysaccharides from the Brown Alga Spatoglossum vietnamense
Source: Molecules. 2024 Oct 22;29(21):4982. doi: 10.3390/molecules29214982 (PMC11548010; doi:10.3390/molecules29214982)
Supplement: Supplementary file 1 [file molecules-29-04982-s001.zip › Figure S2.pdf]

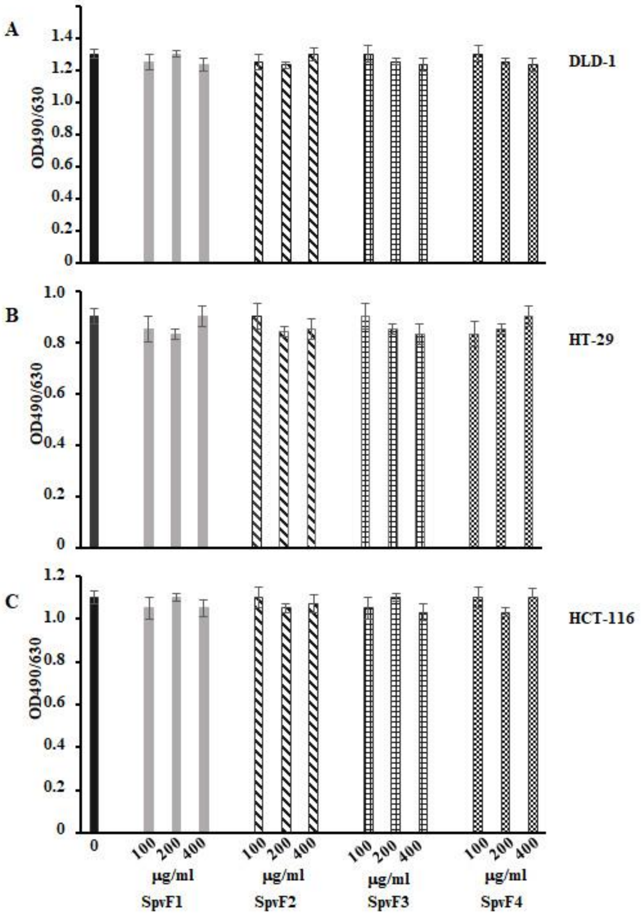

Figure S2. The cytotoxicity of fucoidans SpvF2, SpvF3, SpF4 from *S. vietnamense* against colon cancer cells DLD-1 (A), HT-29 (B), and HCT-116 (C).
