# Supplementary figures and images for: Anticancer Activity In Vitro of Sulfated Polysaccharides from the Brown Alga Spatoglossum vietnamense
Source: Molecules. 2024 Oct 22;29(21):4982. doi: 10.3390/molecules29214982 (PMC11548010; doi:10.3390/molecules29214982)

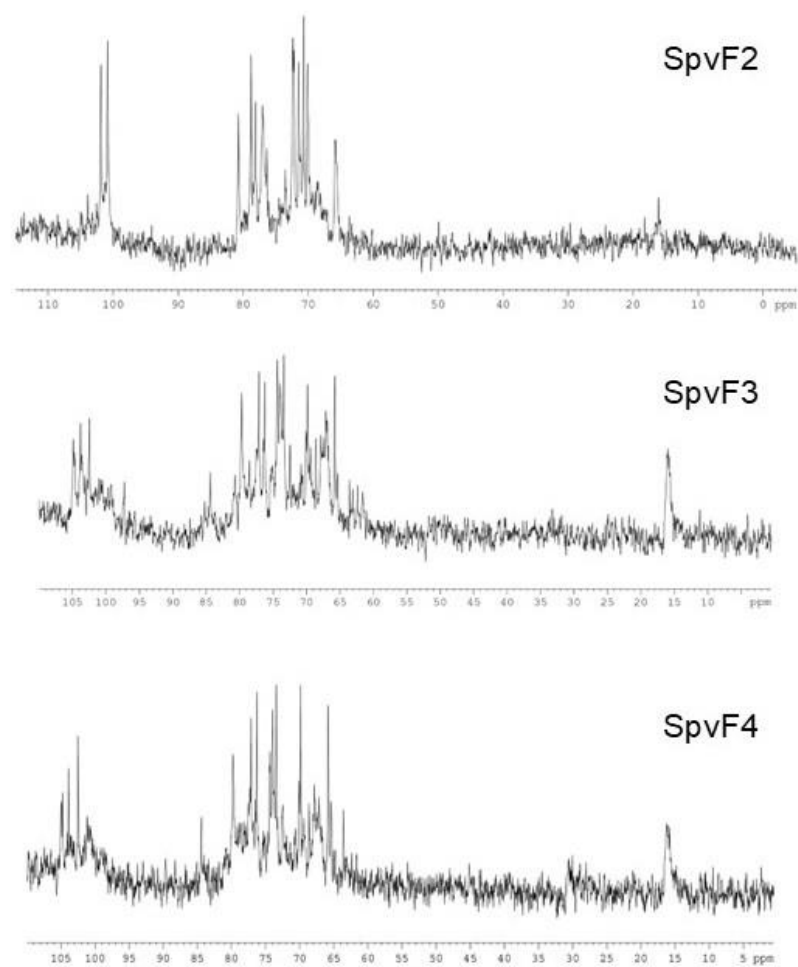

Figure S1. The  $^{13}\text{C}$  NMR spectra of fucoxanthin SpvF2, SpvF3, SpvF4

Supplement: Supplementary file 1 [file molecules-29-04982-s001.zip › Figure S1.pdf]
